# Elbow Flexors Muscle Fat Fraction Is a Sensitive and Relevant Outcome Measure in Nonambulant Patients With DMD

**DOI:** 10.1002/nbm.70306

**Published:** 2026-05-06

**Authors:** M. Michaëls, K. J. Naarding, M. van der Holst, N. Geijsen, E. H. Niks, H. E. Kan

**Affiliations:** ^1^ Department of Neurology Leiden University Medical Center (LUMC) Leiden the Netherlands; ^2^ Duchenne Center Leiden the Netherlands; ^3^ The Novo Nordisk Foundation Center for Stem Cell Medicine (Renew) Leiden the Netherlands; ^4^ Department of Rehabilitation Amsterdam University Medical Center Leiden the Netherlands; ^5^ Department of Orthopedics, Rehabilitation and Physiotherapy Leiden University Medical Center Leiden the Netherlands; ^6^ Department of Anatomy & Embryology Leiden University Medical Center Leiden the Netherlands; ^7^ C.J. Gorter MRI Center, Department of Radiology LUMC Leiden the Netherlands

## Abstract

Duchenne muscular dystrophy (DMD) is an X‐linked neuromuscular disease characterized by progressive muscle wasting. Objective and relevant outcome measures are needed for longitudinal follow‐up and clinical trials. Quantitative MR muscle parameters in the legs are sensitive to change and related to function. However, data are limited for the upper limb, which is crucial for nonambulant DMD patients. This study investigated which MR parameters were sensitive to change over time and associated with loss of upper limb function. Muscle fat fraction (mFF) and contractile volume (CV) for elbow flexor and extensor muscles were obtained using chemical‐shift–based fat‐water separation scans at 3T (Philips, Ingenia) at baseline, 12, and 18 months. Standardized response means (SRM) were determined for different analysis volumes: center slice (CS), 5 (5S), 7 (7S), and all slices (AS). Parameters with SRM > 0.8 were associated with Performance of the Upper Limb, PUL2.0, using linear mixed models. Twenty nonambulant DMD patients participated. Baseline mFF ranged between 43.1% ± 12.6% for elbow extensors AS and 55.5% ± 18.9% for elbow flexors CS. SRM ranged from 0.37 for elbow flexors CV CS to 1.71 for elbow flexors AS and was consistently higher for increasing analysis volumes, longer follow‐up time, and for mFF in comparison with CV. Associations with function were significant for all parameters and ranged from −0.25 to −0.34 (*p* < 0.001) for mFF and 0.12–0.49 (*p* < 0.001) for CV, except for elbow extensor CS CV (*p* = 0.87). Our study demonstrates that assessment of a larger part of the muscle increases the sensitivity to change over time in nonambulant DMD patients. Elbow flexor and extensor mFF 5S, 7S, and AS were sensitive to change over 12 months and related to loss of upper limb function in this population.

## Graphical abstract

Quantitative MRI of upper limb muscles in DMD patients showed increasing sensitivity to change over time with larger analysis volumes and longer follow‐up. Muscle fat fraction had the highest sensitivity to change and was associated with declining upper limb function, supporting its value as a robust imaging biomarker in DMD.

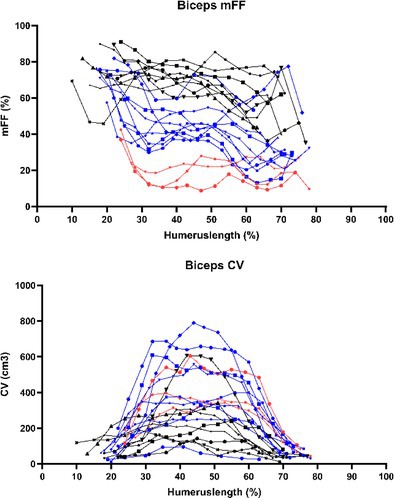

## 1 Introduction

Duchenne muscular dystrophy (DMD) is a rare, X‐linked neuromuscular disease characterized by progressive muscle wasting. Muscle tissue is progressively replaced by fat and fibrotic tissue [1]. The first motor symptoms appear around the age of 2–5 years and are more pronounced in the lower limbs than the upper limbs. DMD patients become wheelchair dependent around the age of 10–12 years, and upper limb function becomes increasingly important for activities of daily living. Patients have a reduced life expectancy of around 20–40 years [2, 3]. There is no cure available, although there are many current and upcoming clinical trials. Multiple outcome measures for muscle strength and function of the upper limb are approved by regulatory bodies for use in these clinical trials [4, 5]. The Performance of the Upper Limb (PUL) module 2.0 [6] is increasingly used as a primary endpoint and assesses the function of the proximal, middle, and distal dimensions of the upper limb. However, patient effort and motivation, as well as the motivational abilities of the clinical evaluator, play a role in these assessments, resulting in variability and larger patient numbers to reach sufficient statistical power. Therefore, there is a need for more sensitive and objective outcome measures that relate to arm function and can be used in longitudinal follow‐up and clinical trials across disease stages.

Quantitative magnetic resonance (MR) muscle parameters are promising biomarkers being less dependent on motivation and objective. For a biomarker to serve as an endpoint, it must be reliable and relate to changes that are relevant for patients, as new outcome measures are approved based on their ability to provide clinically meaningful data while addressing limitations of existing measures (ema.europa.eu). Manual segmentation has been shown to reliably determine muscle volumes from MRI, and accurate volume estimates can still be obtained when fewer, appropriately selected slices are used [7]. Skeletal muscle fat fraction (mFF) has been used most frequently in trials and natural history studies of patients with DMD. In lower limb muscles, it has been shown that mFF can reduce sample size by a factor of 2–7 compared with functional tests due to its high sensitivity to detect change over time [8, 9, 10]. It is associated with muscle function and can be predictive of future clinical milestones [11, 12, 13, 14, 15, 16]. In the upper limb, mFF is associated with muscle function and can even detect changes when functional assessments are still normal [11, 16, 17, 18]. Elbow flexor mFF has an added predictive value for the clinical milestone loss of hand‐to‐mouth movement in nonambulant DMD patients [19].

Importantly, mFF varies along the proximo‐distal axis of the lower limb and forearm muscles [17, 20, 21, 22], which necessitates accurate repositioning or assessment of a large part of the muscle to be reproducible, especially in a growing population like DMD. It is unclear if these proximo‐distal differences are also present in the upper arm and how this affects the sensitivity to change over time. As a result, it is uncertain if assessing a single slice in the middle of the muscles or assessing all slices would yield the highest sensitivity to detect change. Furthermore, as mFF is a measure for the noncontractile fraction of muscle tissue and hence the end stage of disease pathology, it does not give information on the contractile tissue itself. By contrast, the contractile volume (CV) represents preserved muscle tissue and therefore might have a stronger association with muscle function compared with mFF. However, CV shows a higher interpatient variability than mFF in lower limb muscles [20, 23], decreasing the sensitivity to detect change over time within a group, and thereby poses a challenge in clinical trials.

This study investigated which MR parameters were sensitive to change over time and related to upper limb function in nonambulant DMD patients. Specific aims were to (i) describe the proximo‐distal differences in quantitative MR parameters of the upper arm, (ii) investigate which MR parameters are sensitive to change over time, and (iii) associate sensitive parameters with upper limb function in nonambulant DMD patients.

## 2 Methods

### 2.1 Study Design, Strength, and Functional Tests

The medical ethics committee Leiden–Den Haag–Delft (METC‐LDD) approved this study, registered under NL63133.058.17 in the OMON registry. Written informed consent was obtained from patients and their legal representatives prior to enrollment. Twenty nonambulatory DMD patients aged 8 years and older with genetically confirmed DMD diagnosis were included between March 2018 and July 2019, as described previously [19]. Participants visited the Leiden University Medical Center (LUMC) for half a day of assessments of the right arm during three visits: Visit 1, Visit 2 (12 months), and Visit 3 (18 months). Due to the coronavirus disease 2019 (COVID‐19) pandemic, some follow‐up visits were missed. Upper limb function was assessed with the PUL2.0, which includes 22 items and yields a total score ranging from 0 to 42. The assessment is divided into three dimensions: proximal (6 items; score range, 0–12), middle (9 items; 0–17), and distal (7 items; 0–13). A higher score indicates better function [24]. Elbow flexion and extension isometric maximum voluntary contraction strength were assessed with the MicroFET2 handheld dynamometer. MR acquisitions were performed before functional tests to avoid the influence of the functional assessments on the MR signal.

### 2.2 MR Acquisition

**FIGURE 1:**
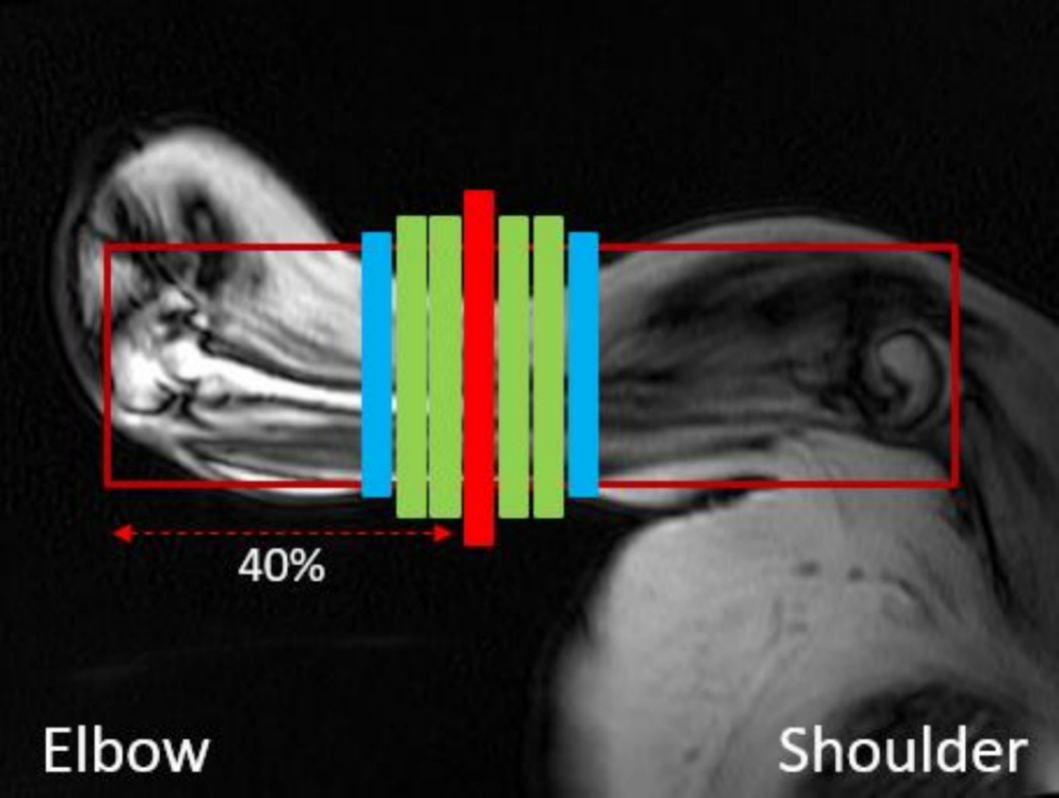
Center slice (CS) is located at 40% humerus length measured from the elbow (red). The 5 center slices include CS with two additional slices on each side (green), and the 7 center slices include an additional slice on each side (blue) compared with 5S.

Upper arm muscles were examined at 3T (Philips Ingenia, Philips Healthcare, Best, the Netherlands) using two circular receive coils of 15 cm placed on the upper arm. Participants were positioned on the right side with the right shoulder and elbow in 90° flexion to have the upper arm muscles as close to bore center as possible. The right hand was supported by a handle placed in a grid on a baseplate matching the MR table width. The handle position was recorded for Visit 1 and used during follow‐up scans to ensure consistent alignment of the upper arm muscles. Only if this position was uncomfortable, a supine position was chosen with the hand in neutral position, between pronation and supination of the forearm. Axial four‐point chemical‐shift–based fat‐water separation gradient echo scans (Dixon) were acquired using a single stack of 33 slices aligned perpendicular to the humeral bone (voxel size, 1 × 1 × 10 mm; repetition time [TR], 310 ms; echo time [TE], 4.40 ms; delta echo time [ΔTE], 0.76 ms; flip angle (FA), 20°; no slice gap; multiacquisition mode; Figure 1).

### 2.3 MR Postprocessing

Water and fat images were generated with in‐house developed software (Matlab 2016a, MathWorks, Natick, MA) using a 6‐peak lipid spectrum; B0 maps were calculated from the phase data of the first and last echoes. Regions of interest (ROIs) were drawn on the muscle border of the biceps, triceps, and brachialis muscles on all slices for every visit using the MIPAV application (Medical Image Processing, Analysis, and Visualization, NIH, Center for Information Technology, version 7.4.0). ROIs from different time points were aligned. The muscle border between the biceps and brachialis muscles was not visible on every slice. Where possible, separate ROIs for each muscle were drawn. For the proximo‐distal analysis, the separate ROIs were used. For the detection of change over time and relation to function, combined ROIs for biceps and brachialis were analyzed, referred to as elbow flexors. The center slice (CS) was defined at 40% humerus length measured from the distal end, as this was anticipated to be the thickest part of the elbow flexor muscles. Slices that contained artifacts or insufficient signal were excluded. Total volume (TV) per muscle was defined as the sum of the respective ROIs times slice thickness (TV = Σ_ROIs_ × slice thickness). ROIs were then eroded by Acquisition 2 voxels to exclude fascia and subcutaneous fat in order to determine mFF. For each ROI, mFF and CV were determined as follows:

$$\mathrm{mFF}=\frac{1.05*\mathrm{Fat}}{1.25*\text{Water}+1.05*\mathrm{Fat}}$$

$$\mathrm{CV}=\mathrm{TV}*\left(1-\mathrm{mFF}\right)$$

with assuming T1 values for water and fat are 1420 ms (correction factor 1.25) and 371 ms (correction factor 1.05) to correct for partial saturation due to T1 effects [25].

### 2.4 Statistical Analysis

Analysis of the data involved three steps: (1) visual assessment of proximo‐distal differences per muscle, (2) assessment of sensitivity to detect change over time, and (3) association of MR parameters to function.

To visualize the differences of mFF along the proximo‐distal axis, mFF was plotted against the relative humerus length to account for different muscle sizes. Muscles were divided into three groups according to mFF at Visit 1 to aid visual inspection: Low mFF was defined as < 30% mFF in the CS, medium mFF as CS between 30% and 60%, and high mFF as CS > 60% fat.

To investigate the effect of proximo‐distal differences in quantitative MR parameters on sensitivity to detect change, we used the longitudinal follow‐up data. We analyzed mFF and CV for different analysis volumes: center slice (CS), 5 center slices (5S), 7 center slices (7S), and all slices (AS) (Figure 1). For multislice assessment, mFF was calculated as a weighted mean value by averaging voxels of included ROIs. The weighted average of all ROIs for that muscle was used for AS mFF. Sensitivity to change over time was determined using the standardized response mean (SRM), and this was calculated for MR parameters mFF and CV for all analysis volumes: AS, 7S, 5S, and CS, for elbow flexors (biceps and brachialis) and elbow extensor (triceps):

$$\mathrm{SRM}=\frac{\text{mean}1-\text{mean}2\;}{\mathrm{SD}\;\text{of the change}}$$

An SRM ≥ 0.8 indicates a large responsiveness to change, 0.5–0.8 a moderate, and < 0.5 a low responsiveness [26]. Corresponding sample sizes (SS) were calculated using Lehr's formula for a potential trial designed to detect a 50% reduction in disease progression (effect size [E]) with 80% power and significance level of *p* < 0.05. SS represents the number of participants per group, assuming equal allocation [27]:

$$\mathrm{SS}=\frac{16\;}{{\left(E*\mathrm{SRM}\right)}^{2}}$$

Linear mixed models were applied to account for repeated measures per patient and to assess the association between MR parameters with SRM > 0.8 and the PUL2.0. The model included one fixed effect, the MR parameter, and one random effect, the participant, for all visits with available data. The reported estimate describes how many units upper limb function changes per unit change of MR parameter, and was considered significant for *p* < 0.05. All statistics were performed in R, v4.3.3, package haven v2.5.3, lme4 v1.1–35, lmertest v3.1–2 (R Core Team 2021).

## 3 Results

Median age was 13.5 (12.5–16.4 years) at first visit. Fifteen patients returned for their 12‐month visit and 11 at 18 months. One patient switched to a medication trial after the first visit and therefore excluded from follow‐up. Three 12‐month and eight 18‐month follow‐up visits were missing due to COVID‐19 restrictions. One follow‐up scan at 12 months was excluded from flexor and extensor analysis due to insufficient signal. Another participant had consistent artifacts during all three visits in the triceps region from the CS to the distal end; all three visits of this participant were excluded from the elbow extensor analysis. Two participants had artefacts in the triceps region at 18 months follow‐up. Scans included 20 flexors and 19 extensors at baseline, 15 and 14 at 12 months, and 11 and eight at 18 months, respectively.

Thirty‐eight scans were performed with patients on their right side and elbow in 90° flexion. Eight scans were performed in supine position with the right arm extended next to the body: two at Visit 1, two at Visit 2, and four at Visit 3 (Supporting Information S1).

### 3.1 Proximo‐Distal Differences

**FIGURE 2:**
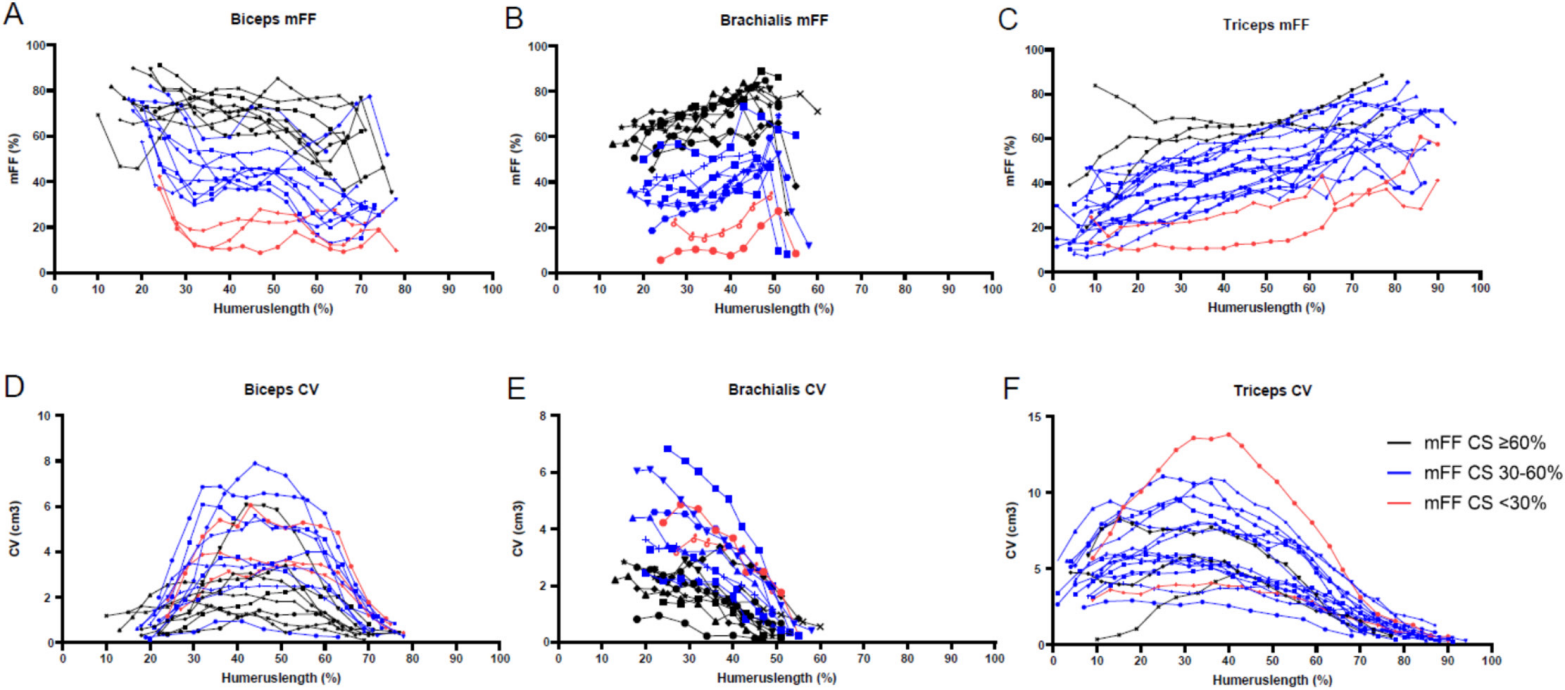
Proximo distal distribution of mFF over the upper arm at Visit 1. mFF is plotted relative to humerus length, with 0% representing the distal region (left side of the graph) and 100% the proximal (right side). Each point represents one slice. Black lines represent patients with mFF ≥ 60% at the center slice (CS); blue lines represent mFF between 30% and 60%, and red lines represent mFF < 30%. (A) mFF of the biceps muscle demonstrates a higher variability at both muscle ends compared with the center region. (B) mFF of the brachialis muscle demonstrates a higher variability, mainly at the proximal muscle end. (C) mFF of the triceps muscle with a consistently higher mFF toward the proximal end. (D) CV of the biceps muscle is larger for slices in the center region and shows gradually smaller CVs toward both muscle ends. (E) CV of the brachialis muscle is largest in the most distal slices, with gradually smaller CVs toward the proximal end of the muscle. The distal portion of the brachialis is not visualized due to the field of view centered on the humerus, whereas brachialis insertion lies distal to the elbow joint. (F) CV of the triceps muscle is larger for slices in the center region and smaller toward both muscle ends.

Upon visual inspection, mFF was not uniformly divided across the proximo‐distal axis of the upper arm muscles. The proximo‐distal differences in mFF were especially clear at both ends of the biceps muscle (Figure 2A) and at the proximal end of the brachialis muscle (Figure 2B). The triceps muscle showed a consistent difference between adjacent slices across the whole proximo‐distal axis (Figure 2C). CVs of the biceps and triceps muscles were largest at the center region of the muscle corresponding to the muscle belly and gradually smaller toward both muscle ends (Figure 2D,E). The CV of the brachialis muscle was largest at the most distal slices (Figure 2F), consistent with its anatomical location beyond the field of view. As a result, the AS analysis did not include the entire brachialis muscle.

The proximo‐distal differences had a small but consistent effect on weighted mFF values between the analysis volumes (Table 1). The mFF was consistently smaller at larger analysis volumes. For instance, CS elbow flexors mFF at Visit 1 was 55.5% and AS mFF was 52.4%. Slice‐by‐slice mFF and CV change from baseline to 12 months showed relatively consistent changes in slices located in the central region of elbow flexors and extensor. Greater variability was observed toward muscle ends, particularly for the elbow flexors compared with the elbow extensor (Supporting Information S3).

**TABLE 1:** Average values and sensitivity to change over time of quantitative upper limb MR parameters, strength measures, and PUL2.0.

Parameters	Visit 1 (*n* = 20)	Visit 2, 12 months (*n* = 15)			Visit 3, 18 months (*n* = 11)		
		Mean change (SD)	SRM	SS	Mean change (SD)	SRM	SS
Muscle fat fraction (%)							
Elbow flexors all slices	52.4 (18.2)	5.4 (4.9)	**1.11**	52	10.0 (5.8)	**1.71**	22
7 center slices	53.7 (18.5)	4.8 (4.7)	**1.01**	63	10.2 (6.1)	**1.68**	23
5 center slices	54.5 (18.3)	4.7 (4.8)	**0.96**	69	9.4 (6.2)	**1.51**	28
Center slice	55.5 (18.9)	3.8 (6.3)	0.60	181	8.7 (7.1)	**1.22**	43
Elbow extensor all slices	43.1 (12.6)	6.5 (5.3)	**1.23**	43	11.7 (7.8)	**1.49**	29
7 center slices	44.4 (13.8)	5.5 (5.3)	**1.03**	61	9.8 (7.7)	**1.28**	40
5 center slices	44.7 (13.8)	5.3 (5.4)	**0.98**	68	9.5 (7.8)	**1.21**	44
Center slice	45.1 (14.0)	5.1 (5.4)	**0.93**	74	9.1 (7.6)	**1.20**	45
Total volume (cm^3^)							
Elbow flexors all slices	118.3 (45.4)	−5.5 (17.3)	—	—	−9.2 (26.4)	—	—
7 center slices	74.3 (26.4)	−6.2 (11.1)			−5.0 (15.3)		
5 center slices	56.1 (20.2)	−4.1 (8.3)			−3.1 (12.2)		
Center slice	11.7 (4.1)	−0.6 (1.7)			−0.5 (0.2)		
Elbow extensor all slices	185.5 (76.4)	−6.8 (19.9)	—	—	−13.7 (32.0)	—	—
7 center slices	81.8 (32.5)	−4.3 (6.1)			−10.0 (13.2)		
5 center slices	59.6 (23.8)	−2.9 (4.7)			−7.2 (10.4)		
Center slice	12.1 (4.8)	−0.6 (1.2)			−1.6 (2.4)		
7 center slices	33.8 (17.3)	−1.6 (4.5)	0.36	504	−5.2 (5.5)	**0.95**	71
5 center slices	25.4 (12.9)	−1.7 (4.0)	0.41	374	−2.9 (5.0)	0.57	200
Center slice	5.1 (2.6)	−0.3 (0.8)	0.37	480	−0.7 (1.0)	0.76	112
Contractile volume (cm^3^)							
Elbow flexors all slices	55.0 (27.9)	−5.0 (7.0)	0.71	126	−8.0 (7.6)	**1.04**	59
Elbow extensor all slices	101.0 (39.9)	−9.1 (17.3)	0.53	233	−18.6 (17.8)	**1.05**	59
7 center slices	43.5 (17.4)	−2.8 (4.9)	0.57	196	−5.7 (5.2)	**1.09**	55
5 center slices	31.5 (12.7)	−2.0 (3.4)	0.57	195	−3.9 (3.8)	**1.02**	62
Center slice	6.4 (2.6)	−0.4 (0.7)	0.55	217	−0.7 (0.7)	**0.95**	71
Functional parameter							
PUL2.0 (Visit 1: Median–IQR)	21 (19–34)	–3 (2.36)	**1.27**	40	−4 (3.41)	**1.17**	47
Elbow flexion strength, *N*	19.3 (12.3)	−0.87 (3.6)	0.21	1387	−3.85 (3.5)	**1.10**	53
Elbow extension strength, *N*	20.9 (15.8)	−2.35 (4.2)	0.56	204	−2.44 (4.4)	0.56	207

*Note:* Mean values reported or specified if different. SRM values ≥ 0.8 are considered sensitive to change and shown in bold.

Abbreviations: CV = contractile volume, mFF = muscle fat fraction, N = Newton, PUL2.0 = Performance of the Upper Limb module 2.0, SRM = standardized response mean, SS = sample size per group.

### 3.2 Sensitivity to Change

**FIGURE 3:**
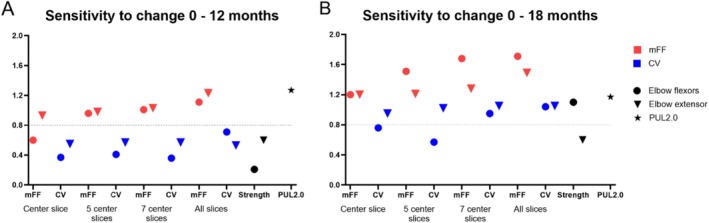
Standardized response mean values for elbow flexor and extensor muscle fat fractions (mFF) and contractile volumes (CV) for center slice, 5 center slices, 7 center slices, and all slices are visualized next to the SRM of elbow flexion and extension strength and the PUL2.0 in (A) elbow flexors and extensor 0–12 months and (B) elbow flexors and extensor 0–18 months. The dashed line at the SRM value of 0.8 represents the threshold, above which a parameter is considered to be sensitive to change.

We next assessed the sensitivity to detect change for all variables. The SRM for mFF was consistently higher for larger analysis volumes; where the CS for elbow flexor mFF showed an SRM of 0.60 at 12 months, below the 0.8 threshold, the SRM values for elbow flexor mFF for 5S, 7S, and AS were all above 0.8 and thus considered to be sensitive to change over 12 months. As expected, SRMs also increased over time and were higher at 18 months compared with 12 months (Table 1 and Figure 3). mFF SRMs were consistently higher than those for CV (Figure 3). At 18 months, all mFF values demonstrated SRMs above 0.8. Elbow flexor CV reached an SRM above 0.8 only for AS and 7S, whereas extensor CV reached were all above 0.8 at 18 months. In contrast, all strength SRMs were below 0.8, except for elbow flexor strength at 18 months. The SRM of PUL was 1.27 at 12 months and 1.17 at 18 months. The highest SRM of 1.71 was for elbow flexor mFF of all slices at 18 months follow‐up, corresponding to a required number of 22 participants per group. By contrast, when using the center slice, almost double the number of participants (43 per group) would be required. This effect is even stronger at the 12‐month interval, where SS reduces from 181 for CS to 52 for AS (Table 1).

### 3.3 Association to Function

The elbow flexor and extensor mFF of all analysis volumes, except flexor CS, had an SRM ≥ 0.8 and were associated with PUL2.0. The mixed models showed an estimate of −0.3 for elbow flexor mFF of AS, meaning that a 1 point increase in weighted fat fraction of all slices related to a decrease of 0.3 points on the PUL2.0 (Table 2). Elbow flexor mFF for 7S, 5S, and CS showed similar estimates, indicating that a change in mFF relates to a change in PUL2.0 to the same degree for every analysis volume.

**TABLE 2:** Association of MR parameters with the functional PUL2.0 outcome measures.

Parameters	LMM estimates	*p*
Elbow flexors mFF (%) all slices	−0.32	< 0.0001
7 center slices	−0.33	< 0.0001
5 center slices	−0.34	< 0.0001
Center slice	−0.32	< 0.0001
Elbow extensor mFF (%) all slices	−0.25	0.0005
7 center slices	−0.32	< 0.0001
5 center slices	−0.33	< 0.0001
Center slice	−0.33	< 0.0001
Elbow flexors CV (cm3) all slices	0.28	< 0.0001
7 center slices	0.42	< 0.0001
Elbow extensor CV (cm3) all slices	0.12	0.0002
7 center slices	0.36	< 0.0001
5 center slices	0.49	< 0.0001
Center slice	−0.05	0.87

*Note:* MR parameters that were sensitive to change over 18 months (SRM ≥ 0.8) were associated with PUL2.0. The estimate corresponds to a change of PUL2.0 score for a 1‐unit change in the MR parameter.

Abbreviations: CV = contractile volume, LMM = linear mixed model, mFF = muscle fat fraction, PUL2.0 = Performance of the Upper Limb module 2.0.

CV of the elbow flexors for AS and 7S and for all extensor analyses volumes had an SRM ≥ 0.8 and were associated with PUL2.0. The effect was larger at 7S compared with AS: a 1 point increase in CV of 7S and AS analysis volumes related to an increase of respectively 0.4 points and 0.3 points on the PUL2.0. The effect of elbow flexor CV was larger than that of the elbow extensor: The PUL2.0 score declined by 0.3 points for a 1‐unit decline in flexor CV for AS and only 0.1 points for a 1‐unit decline in extensor CV (Table 2).

## 4 Discussion

In this study, we aimed to assess the proximo‐distal differences, sensitivity to change over time, and the relationships to upper limb function of several quantitative MR parameters of the upper arm. We showed that upon visual inspection, mFF was not uniformly distributed across the proximo‐distal axis of the upper arm muscles. In addition, we showed differences in SRM between analysis volumes, where the largest difference was between single‐slice and larger analysis volumes. Of the qMR parameters assessed, mFF consistently showed higher sensitivity to detect change over time than CV, showing a greater potential to reduce sample sizes in clinical trials. Lastly, all sensitive MR parameters were related to upper limb function.

Our findings of proximo‐distal differences of mFF in upper arm muscles are in agreement with previous studies of the lower limb and forearm muscles, and highlight the importance of analyzing a larger volume to capture spatial differences. The fat‐fraction distribution patterns differ across muscles, ranging from a higher mFF in proximal and distal regions compared with the central part to predominantly higher mFF distally [17, 20, 21, 22]. In the upper arm, we observed a lower mFF proximally in the biceps, whereas the brachialis and triceps muscles showed lower mFF distally. Additionally, the most proximal slice of the brachialis showed an exceptionally low mFF. The low values in the proximal part of the biceps and most proximal slice of the brachialis may be attributed to small ROI sizes and increased tendon tissue present near muscle ends, resulting in a higher variability. Additionally, intermuscle differences in mFF were observed, with the elbow flexors typically having a 10% higher mFF than extensors, consistent with previous findings for upper arm muscles in DMD [17, 18, 28]. Overall, the difference in elbow flexor mFF between the CS and AS of 3.2% is comparable with annual changes in mFF of between 3.8 (SD 6.3%) to 5.4 (4.9%) in our population and comparable with previous reported mean annual changes on upper limb values of 7.0% [29] and lower limb values of 3.0%–7.0% depending on the muscle [8, 10, 22]. These findings underscore the sensitivity of mFF measurements to repositioning errors, particularly for small analysis volumes, and show that a fixed reference point—such as 40% humerus length—may increase reproducibility.

Our results show that the mFF of upper arm muscles is sensitive to detect change over 12 months in nonambulant DMD patients, with SRM values ranging from 0.60 to 1.11. This is in line with previous findings for mFF in muscles of the lower limb for ambulatory patients (SRM: 0.32–1.25) and upper limb for nonambulatory patients (SRM: 0.50–1.40) [30, 31, 32]. The higher sensitivity to detect change for mFF compared with CV is in line with previous findings in DMD [31] and other muscular dystrophies, like Becker muscular dystrophy [9], LGMD R9 [33], and dysferlinopathy [34]. This difference likely stems from the fact that mFF is constrained to a fixed range of 0% and 100%, whereas muscle volume and therefore CV can vary considerably more between different individuals and are subject to maturation in a pediatric population. This also complicates the comparison between the calculated sample sizes and the calculation with Lehr's formula. In this formula, the same effect of slowing down disease progression with 50% is assumed for both outcome measures, whereas CV might increase due to growth. This could have led to an overestimation of the SS for CV. Sensitivity to detect change over time increased with larger analysis volumes, indicating that larger analysis volumes are preferable over smaller volumes. However, selecting an optimal analysis volume is challenging. Especially in a growing population, such as DMD, whole muscle analysis may better account for growth but presents challenges. It leads to increased analysis time due to the number of ROIs to be drawn, to increased difficulty in discerning the muscles from each other at the proximal and distal ends, and to increased acquisition time. In our setup, the distal region was more prone to artefacts due to the tissue variations at the elbow joint, exacerbated with patient positioning (right side, shoulder, and elbow in 90° flexion), resulting in the elbow displaced outside the bore center along the x‐axis in the majority of the participants. A fixed volume approach, such as 5S or 7S, has been used in previous DMD studies [10, 35] and other neuromuscular diseases as well [9, 36]. In our data, the difference in SRMs of the fixed analysis volumes was small, and all provided sensitive MR parameters for a follow‐up period of 12 and 18 months. Therefore, a fixed volume (multislice) approach is preferable as it is practical and robust in the upper arm muscles and encompasses a larger part of the muscle while excluding regions prone to artifacts.

To serve as an outcome measure, MR parameters should also relate to changes that are relevant for patients. Especially, the elbow flexors play an essential role in upper limb tasks, such as the hand‐to‐mouth movement necessary for feeding and personal care, whereas elbow extensors are important for reach. Recent qualitative research has shown that even small changes in upper limb function, particularly those affecting independence in ADL, are perceived by patients and caregivers as highly meaningful [37]. In our dataset, elbow flexor and extensor mFF of 5S, 7S, and AS were all associated with PUL2.0, with the estimated effect of 1% increase in fat fraction related to a loss of 0.3 points on the PUL2.0 total score, indicating that they are relevant outcome measures for our nonambulant DMD patients. This is in agreement with previous findings relating forearm mFF to PUL [22].

Our study focused on nonambulant DMD patients, based on the expectation that this group would show the greatest decline in upper limb function within the duration of the study. As a result, mFF in this group was already around 50% at baseline, indicating relatively advanced disease. Quantitative MR parameters may be particularly valuable at earlier disease stages, when functional decline is still subtle and functional outcome measures lack sensitivity to change over time due to ceiling effects. In such cases, MRI can detect underlying muscle changes before measurable functional loss and predict loss of functional milestones [13, 18, 23, 38]. This enables earlier intervention and more precise monitoring of therapeutic effects across disease stages.

Limitations of this study include the small sample size and the loss to follow‐up at 18 months compared with 12 months. Although this may have influenced the sensitivity to change over time (Supporting Information S2), MR parameters were shown to be sensitive to change over time and relevant for our patients. We used Lehr's formula to simplify the interpretation for potential clinical trials. This assumes 50% reduction in disease progression for all outcome measures, but in a growing population, this might have led to an underestimation of the SRM and overestimation of SS for CV. Also, the positioning in the scanner turned out to be prone to artifacts at the distal end of the upper arm and challenging for some patients. A supine position with the extended arms positioned next to the body could help in consistent positioning for longitudinal assessments in the ambulant and nonambulant stage.

In conclusion, proximal‐distal differences of MR parameters are present in the upper limb of nonambulant DMD patients. The analysis volume has an influence on the sensitivity to detect change over time, where the largest difference was between single‐slice and larger analysis volumes. A fixed volume approach covering a large part of the muscle is thus preferable as it avoids regions prone to artefacts while limiting scan and analysis time. Finally, elbow flexor and extensor mFF for 5 or 7 center slices are related to relevant functional changes in our nonambulant DMD population and reduce the required SS needed for a clinical trial over a period of 18 months to around 25 patients compared with 47 patients using PUL, highlighting its potential as an outcome measure in clinical trials.

## Author Contributions

**Michel Michaëls:** data analysis, writing manuscript. **Karin J. Naarding:** study design, data collection, data analysis, reviewing manuscript. **Menno van der Holst:** data collection, reviewing manuscript. **Niels Geijssen:** reviewing manuscript. **Erik H. Niks:** study design, data analysis, reviewing manuscript. **Hermien E. Kan:** study design, data analysis, reviewing manuscript.

## Funding

This work was partially funded by the Novo Nordisk Foundation Center for Stem Cell Medicine, supported by a Novo Nordisk Foundation grant NNF21CC0073729, and partially funded by the Stichting Spieren voor Spieren grant SvS15.

## Conflicts of Interest

The authors declare no conflicts of interest.

## Supporting information


**Data S1:** Supporting information.

## Data Availability

The data that support the findings of this study are available from the corresponding author upon reasonable request.
