# Supplementary material for: Elbow Flexors Muscle Fat Fraction Is a Sensitive and Relevant Outcome Measure in Nonambulant Patients With DMD
Source: NMR Biomed. 2026 May 6;39:e70306. doi: 10.1002/nbm.70306 (PMC13148194; doi:10.1002/nbm.70306)
Supplement: Supplementary file 1 — Data S1: Supporting information. [file NBM-39-e70306-s001.docx]

Supplemental data.

**S1. Alternative positioning, excluded and missing data from analyses**

| **Parameters** | **First visit (n=20)** | | **12 months visit (n=15)** | | **18 months visit (n=11)** | |
| --- | --- | --- | --- | --- | --- | --- |
|  | **Elbow flexors** | **Elbow extensor** | **Elbow flexors** | **Elbow extensor** | **Elbow flexors** | **Elbow extensor** |
| DMDUE006 |  |  |  |  |  |  |
| DMDUE007 |  |  |  |  |  |  |
| DMDUE011 |  |  |  |  |  |  |
| DMDUE012 |  |  |  |  |  |  |
| DMDUE016 |  |  |  |  | missed | missed |
| DMDUE018 |  |  |  |  | missed | missed |
| DMDUE019 |  |  |  |  | Straight arm | Straight arm |
| DMDUE020 |  |  | Straight arm | Straight arm | Straight arm | Straight arm |
| DMDUE021 |  |  | switched | switched | switched | switched |
| DMDUE022 |  |  |  |  |  |  |
| DMDUE024 |  |  |  |  | missed | missed |
| DMDUE025 | Straight arm | Straight arm |  |  | missed | missed |
| DMDUE026 |  |  |  |  |  |  |
| DMDUE028 | Straight arm | Artifacts CS to distal | Straight arm | Artifacts CS to distal | Straight arm | Artifacts all ROIs |
| DMDUE030 |  |  |  |  | missed | missed |
| DMDUE031 |  |  | missed | missed | missed | missed |
| DMDUE032 |  |  | Insufficient signal | | Straight arm | Artefacts all ROIs |
| DMDUE034 |  |  | missed | missed | missed | missed |
| DMDUE035 |  |  | missed | missed | missed | missed |
| DMDUE039 |  |  |  |  |  | Artefacts all ROIs |

Abbreviations: CS = center slice, ROI = region of interest. Missing data of 11 missed visit were due to COVID-19 restrictions. An alternative ‘Straight arm’ position was used for 8 scans due to discomfort with standard position on the right side (shoulder anteflexion and elbow flexion of 90 degrees). One participant switched to a medication trial between baseline and 12 months. Slices with artefacts were excluded, resulting in the exclusion of slices distal from CS for extensor analysis in two scans and exclusion of all slices from extensor analysis in two scans. One scan was entirely excluded due to insufficient signal for quantitative analysis.

**S2. Sensitivity to change over time of 7S mFF for patients with all three visits**

| **Parameters** | **Visit 1** | **Visit 2, 12 months** | | | **Visit 3, 18-months** | | |
| --- | --- | --- | --- | --- | --- | --- | --- |
|  |  | **Mean change (SD)** | **SRM** | **SS** | **Mean change (SD)** | **SRM** | **SS** |
| **Muscle fat fraction (%)** | | | | | | | |
| Elbow flexors 7S (all data)  Elbow flexor 7S (n=10) | 53.7 (18.5)  56.4 (17.9) | 4.8 (4.7)  7.0 (5.4) | 1.01  1.30 | 63  38 | 10.2 (6.1)  9.8 (6.3) | 1.68  1.55 | 23  27 |
| Elbow extensor 7S (all data)  Elbow extensor 7S (N=10) | 44.4 (13.8)  51.0 (4.3) | 5.5 (5.3)  8.3 (5.8) | 1.03  1.44 | 61  31 | 9.8 (7.7)  11.7 (7.3) | 1.28  1.60 | 40  26 |

Abbreviations: SRM = standardized response mean, SS = Sample Size per group, mFF = muscle fat fraction, CV = contractile volume. Mean values reported or specified if different. SRM values ≥ 0.8 are considered sensitive to change over time.

**S3. Slice by slice delta per subject**


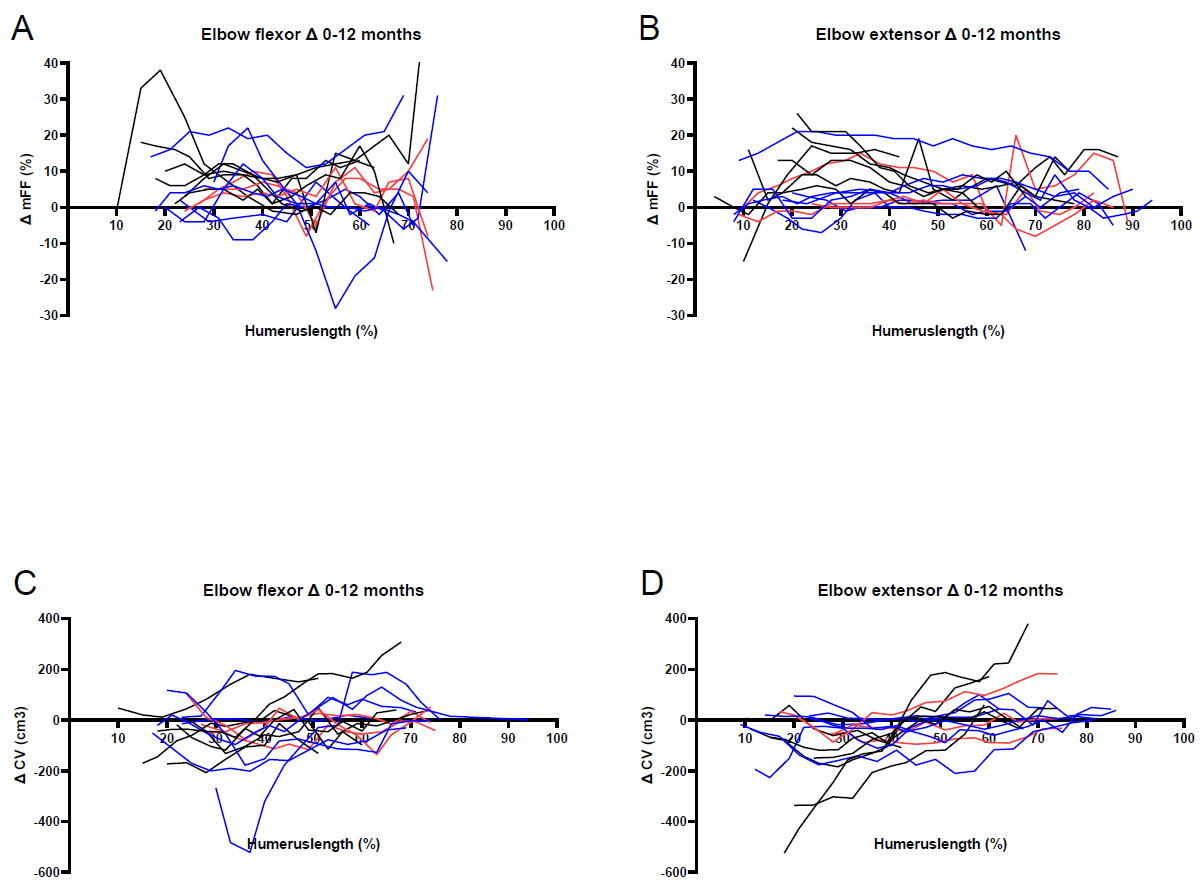


**S2.** Slice by slice delta over time plotted over percentage of humerus length (0% elbow - 100% shoulder) for A: Elbow flexor muscle fat fraction (mFF), B: Elbow extensor muscle fat fraction, C: Elbow flexor contractile volume (CV), D: Elbow extensor contractile volume. Positive value represents an increase from baseline to 12 months, negative value a decrease. Black lines represent patients with baseline mFF ≥60% at the center slice (CS); Blue lines baseline mFF between 30 and 60% and red lines baseline mFF <30%.
